# The mediation of the dynamic capacity for innovation between managerial skills and organizational performance in MSMEs located in the department of Caquetá, Colombia

**DOI:** 10.1371/journal.pone.0312172

**Published:** 2024-10-24

**Authors:** Fernando Penagos Guzman, Mónica García Solarte

**Affiliations:** 1 University of Amazonia, Florencia, Colombia; 2 University of Valle, Cali, Colombia; Zhejiang Normal University, CHINA

## Abstract

**Objectives:**

The purpose of this study is to analyze how the dynamic capacity for innovation mediates the relationship between the managerial skills and organizational performance of micro, small and medium-sized enterprises (MSMEs) located in the department of Caquetá.

**Methods:**

The hypotheses are statistically tested via structural equation modeling (SEM), where the dynamic capacity for innovation mediates the relationship between the managerial skills and organizational performance of MSMEs located in the department of Caquetá, with a cross-sectional sample of 496 MSMEs.

**Results:**

The results indicate that the relationship between managerial skills and organizational performance is mediated by the dynamic capacity for innovation of the MSMEs of the department of Caquetá, Colombia. In addition, the robust adjustment values obtained for this model are an RMSEA of 0.044 and a CFI of 0.862. Both values meet the requirements to conclude that the model has a good fit and is therefore reliable.

**Conclusions:**

This study shows that managers, administrators or legal representatives use the constructs presented in this publication. In addition, it is shown that the dynamic capacity for innovation mediates the relationship between managerial skills and the organizational performance of MSMEs located in the department of Caquetá, Colombia.

## Introduction

This research refers to the incidence rate of managerial skills, organizational performance and the dynamic capacity for innovation in micro, small and medium-sized enterprises (MSMEs) located in the department of Caquetá, Colombia. The influence of managerial skills on organizational performance is assumed to be a factor that generates value in SMEs. The dynamic capacity for innovation is established as a mediating variable between the relationship between managerial skills and organizational performance. In this relationship, it is necessary to determine the role of the manager and his or her management in visualizing the organization as more than a structure in which different internal and external factors that interfere in business development interact.

One of the most significant contributions of this research is based on the environment in which it is carried out and the type of companies analyzed; our study population consists of the MSMEs located in the department of Caquetá. From this perspective, it is considered relevant for the orientation of the current study to consider aspects of these organizations starting from their internal and external variables and the characteristics of their leaders.

Sources used herein are translated using www.DeepL.com/Translator (free version) [1]. Furthermore, for the development of the study, it is necessary to determine aspects such as managerial ability, flexibility and responsiveness, as well as the creation of opportunities, all of which influence MSMEs [2].

MSMEs can be seen as important given their weight in the productive fabric of the region (99% of formal Latin American companies are MSMEs) and in the employment field (61%). This reality makes MSME’s generators of a new development dynamic that allows rapid and continuous economic growth that is simultaneously sustainable [3].

In this sense, it is necessary to discuss the first relationship between the analyzed variables, which refers to the influence of managerial skills on organizational performance, as well as the incidence rate of the dynamic capacity for innovation.

### Managerial skills and their influence on organizational performance

Managerial skills relate to the manager’s ability to direct, organize, motivate and execute different organizational and human relations activities. Therefore, if better organizational performance is sought, then managerial skills are required to promote the optimal development of organizations, for which a dynamic and updated management team with change capabilities is necessary [4]. According to Hernández-Perlines and Ibarra-Cisneros [5], the satisfactory performance of organizations depends on the managerial skills that are immersed in management. In this sense, Coaquira [6] explains that better organizational performance is generated from quality management, knowledge management and leadership, and skills developed by the manager. For this reason, it is necessary to both identify and enhance the managerial skills that impact organizational performance. Moreover, factors such as technology, research, teamwork, and the relationship between internal skills and leadership in the execution of activities add to the process of change for organizational success [7].

One of the most representative skills in performance is strategic leadership, through which the transformation toward innovation is achieved at higher levels in management and organizational activities [8,9].

With respect to the influence of managerial skills on organizational performance, different factors arise in relation to social behaviors and the type of leadership of managers, which represents a broad topic of analysis and discussion. Thus, establishing such dynamics in MSMEs located in the department of Caquetá is relevant for establishing the first working hypothesis, which is stated as follows:

Hypothesis 1: Managerial skills have a positive influence on the organizational performance of MSMEs located in the department of Caquetá.

There is also a relationship between managerial skills and the dynamic innovation capacity, which is discussed below.

### Managerial skills and their impact on the dynamic capacity for innovation

Numerous extant studies have affirmed that the primary effects of the dynamic capacity for innovation occur at the level of organizational processes; therefore, understanding how these capabilities impact business performance is essential [10,11]. The dynamic capacity for innovation is developed at the organizational and administrative levels on the basis of managerial skills [12]. Management skills are an important factor for the development of dynamic innovation capacity, allowing not only good results to be achieved but also resources to be managed correctly and making innovation a must for all functional areas [13]. Notably, the training of managers depends on their ability to develop their skills and, in turn, their willingness to work in innovation processes.

Company managers play the main role in the success or failure of organizations [14]. Enterprises serve as the actors involved in fostering performance units, economic gains and competitive advantages [15]. Therefore, competitive advantage is evidenced as a reflection of the manager’s skills supported by the capability and commitment of the workforce [16]. Although different studies have been conducted to identify the development of management skills of greatest importance to managers [17–19], agreement has not yet been reached, especially on those skills that relate to dynamic innovation capacity [20]. This lack of consensus is due to the environment in which the organizations are subjected, which is changing, dynamic and subject to not only individual perceptions but also the relationships between the subject and the organization [21]. Employee attitudes, leadership styles, recognition, motivation, and job satisfaction, among others, are accounted for [22]. The relationship between managerial skills and the dynamic capacity for innovation arises from environments subject to perceptions, attitudes, behaviors, and social and economic dynamics of an organization. Therefore, it is pertinent to investigate key aspects of these dynamics in MSMEs located in the department of Caquetá. Thus, the second hypothesis of this research arises as follows:

Hypothesis 2: Managerial skills have a positive effect on the dynamic capacity for innovation.

### Dynamic capacity as a mediating variable between managerial skills and organizational performance

From this perspective, it is necessary to use dynamic capacity as a mediating variable between managerial skills and organizational performance. According to [23], dynamic innovation capability both shapes and manages other capabilities in the organization, enabling firms to integrate capabilities and resources. However, few scholars have accurately assessed the role of dynamic innovation capability and its relationship to either managerial skills or organizational performance (an example of a study that uses this approach is Abdel Gawad et al. [24]); rather, studies on this subject have placed special emphasis on the dynamic capacity for innovation in products or processes and not as an organizational variable [25]. In this sense, for the development and optimization of the dynamic capacity for innovation in an organization, it is necessary to coordinate the actions of the manager, which contributes to better organizational performance [26]. Hence, the importance of the relationship between the dynamic capacity for innovation as a mediating variable of managerial skills and its relationship with the performance of SMEs is important.

Thus, the academic discussion revolves around the dynamic capacity for innovation in SMEs. Crossan and Apaydin [27] refer to a multidimensional framework of organizational innovation, linking leadership, innovation and improved performance results. However, in the extensive review on this topic, most references are focused on innovation and not on the importance of dynamic innovation capability in SMEs [28]. Therefore, a review of this topic is needed since the dynamic capacity for innovation is a multifactorial and differential construct in the performance of SMEs [29,30]. Thus, we propose the following working hypothesis:

Hypothesis 3: Dynamic innovativeness mediates the relationship between the managerial skills and organizational performance of MSMEs.

## Methodology

### Sample and data collection

Based on the established delimitations and the unit of analysis being MSMEs located in the department of Caquetá, the current research was carried out in a wide universe to determine the relationships among the constructs of managerial skills, organizational performance and dynamic capacity for innovation in the management of MSMEs. Therefore, the selected study population was based on the total number of MSMEs with legal and natural persons registered and renewed according to their asset value from January 1, 2020, to September 7, 2021, via the Chamber of Commerce of Florencia in Caquetá. Segments were delineated according to the economic activity they carry out; their distribution is as shown in Table 1.

**Table 1:** Division of MSMEs according to economic activity in Caquetá.

Sector	Distribution by activity	Micro	Small	Medium	Large	Total
**A**	Agriculture, Livestock, Hunting, Forestry, and Fishing	286	15	0	0	301
**B**	Mining and Quarrying	24	6	2	0	32
**C**	Manufacturing Industries	1096	14	2	0	1112
**D**	Supply of Electricity, Gas, Steam, and Air Conditioning	10	2	0	1	13
**E**	Water Distribution, Evacuation and Treatment of Wastewater, Waste Management, and Environmental Sanitation Activities	51	11	2	1	65
**F**	Construction	428	54	7	0	489
**G**	Wholesale and Retail Trade, Repair of Motor Vehicles and Motorcycles	7763	141	18	1	7923
**H**	Transportation and Storage	253	22	2	0	277
**I**	Accommodation and Food Services	2069	9	0	0	2078
**J**	Information and Communications	285	3	1	0	289
**K**	Financial and Insurance Activities	87	2	1	0	90
**L**	Real Estate Activities	64	13	1	0	78
**M**	Professional, Scientific, and Technical Activities	478	19	1	0	498
**N**	Administrative and Support Services Activities	297	5	0	0	302
**O**	Public Administration and Defense, Mandatory Social Security Plans	3	0	0	0	3
**P**	Education	50	4	0	0	54
**Q**	Human Health and Social Work Activities	174	15	4	0	193
**R**	Artistic, Entertainment, and Recreational Activities	459	3	1	0	463
**S**	Other Service Activities	2029	37	4	0	2070
**T**	Activities of Households as Employers	1	0	0	0	1
**U**	Activities of Organizations and Extraterritorial Entities	0	0	0	0	0
**Z**	Unclassified	9	0	0	0	9
**Total**		**15916**	**375**	**46**	**3**	**16340**

Legend: Data taken over a range from 1 January 2019 to 7 September 2021.

Source: Florencia Chamber of Commerce in Caquetá (2019).

Similarly, according to the data provided by the Chamber of Commerce of Florencia in Caquetá, the sector to which each of the MSMEs in the department belong is listed in Table 2.

**Table 2:** Division of MSMEs according to sectors of the Caquetá economy.

Sector	Small business	Medium business	Microbusiness	Large business	General total
**Trade**	166	25	7894	1	8086
**Manufacture**	23	2	1198		1223
**Services**	224	19	6786	2	7031
**Grand Total**	**413**	**46**	**15878**	**3**	**16340**

Source: Authors.

### Sample

A total of 604 surveys were obtained, to which filters related to the number of employees were applied; those MSMEs that had fewer than three employees were discarded, resulting in a total of 503 MSMEs. When counting the MSMEs, we identified that some companies had repeated the survey; therefore, these responses were discarded, resulting in a total of 500. In addition, three companies responded to the instrument that did not belong to the department of Caquetá (established in the municipalities of San José de Isnos, Neiva and Bogotá); finally, one additional company was eliminated because it had more than 230 employees, resulting in a total of 496 MSMEs.

### Variables

For the development of the field work instrument used in the present study, a Likert scale questionnaire approach was used, which provided a quantifiable value to the study variables. This approach was applied to a research instrument composed of 44 variables that used a 5-point Likert-type ordinal scale, where 1 was equivalent to totally disagree and 5 was equivalent to totally agree. The distribution of the questions was structured as shown in Table 3.

**Table 3:** Items of the research instrument with their variables.

Construct	Dimension	Variables
**Management skills**	Conceptual skills	6 items
** **	Human skills	6 items
** **	Technical skills	6 items
**Organizational performance**	Inventory internal processes	3 items
** **	Open system inventory	3 items
** **	Rational Model Inventory	3 items
** **	Human Relationship Inventory	3 items
**Dynamic innovation capacity**	Technology options	6 items
** **	Concepts (conceptualization)	3 items
** **	Coproduce (orchestrate)	2 items
** **	Scale (stretch)	3 items
**Total**	** **	**44 items**

Source: Authors.

### Research design

The purpose of this study was to determine the effects of managerial skills on organizational performance, with the mediating factor being the dynamic capacity for innovation. Given that the study involves three factors, it was necessary to apply the Sobel test, which is generally used to measure effects when there is a third variable, i.e., significance, significance, and significance [31].

Therefore, Hypothesis 3 examines the same approach as that outlined in Hypothesis 1 but adds one more factor as a mediator, namely, dynamic innovation capability. This mediation is possible because the correlation coefficients obtained in Hypothesis 2 are significant, i.e., the effects of managerial skills on dynamic innovativeness and the effects of dynamic innovativeness on organizational performance.

### Confirmatory factor analysis

**Fig 1:**
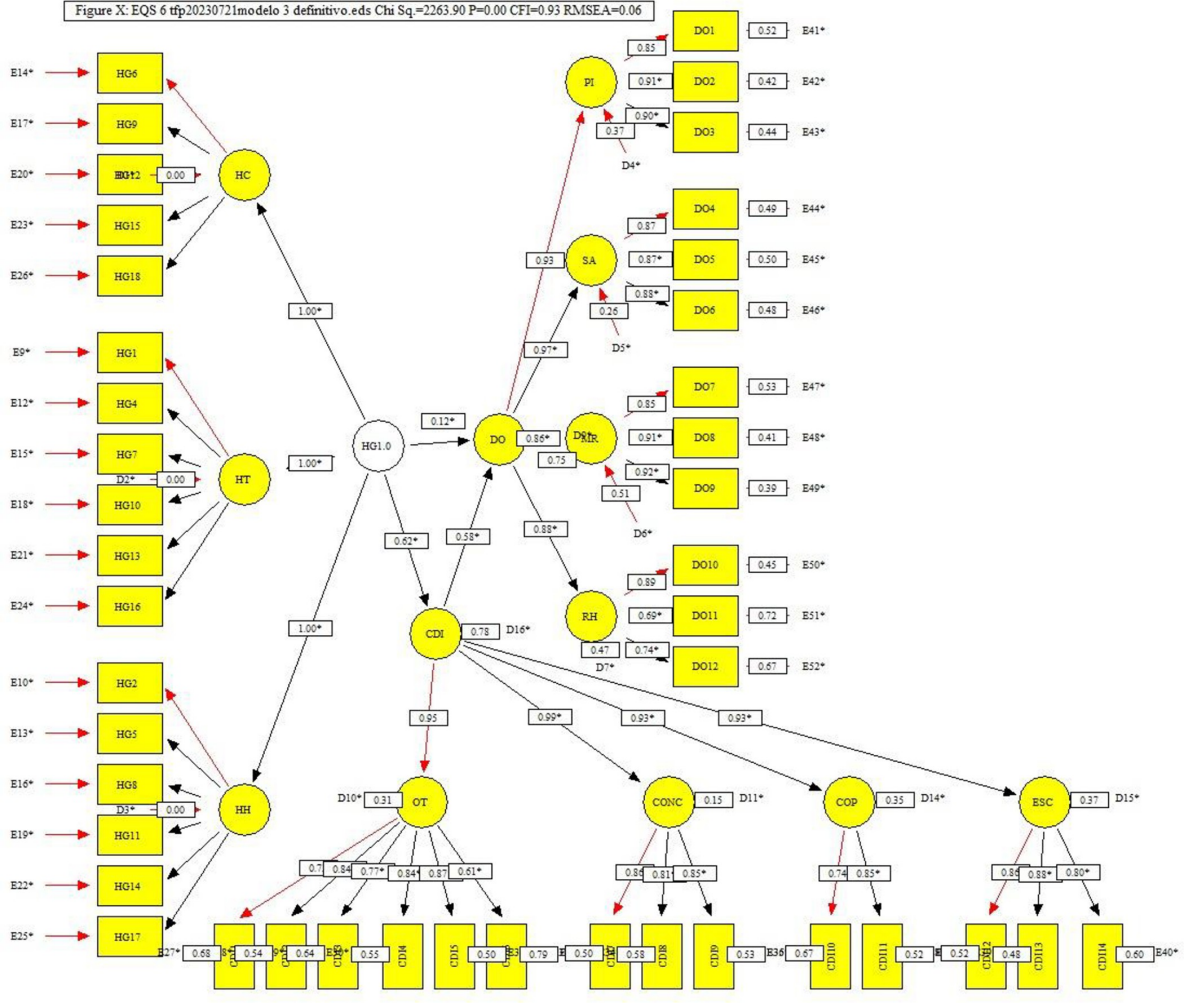
Effects of managerial skills on dynamic innovation capability and the effects of dynamic innovation capability on organizational performance obtained via EQS software. Source: [33] Authors.

We performed a confirmatory factor analysis (CFA) to analyze and evaluate the reliability and validity of the measurement scales used in the theoretical model via structural equation analysis within EQS software version 6.4 by Dr. Peter M. Bentler y Eric Wu for Windows. The reliability of the model was obtained considering Cronbach’s alpha coefficient and the composite reliability index (CRI) [32]. The structural model obtained via EQS software for Model 3 is shown in Fig 1.

### Validation of variables

To measure the reliability and internal consistency of the measurement instrument, that is, the construct validity, the use of Cronbach’s alpha coefficient is recommended. The Cronbach’s alpha value requires compliance with some acceptance conditions such that, according to the proposal of Frías-Navarro [34], if the result is above 0.7, then the construct or instrument is considered acceptable and, therefore, reliable for validation. However, for research in the experimental phase or early stages of analysis, for the purpose of designing new instruments, as well as research projects, it is permissible to continue with the study regardless of the references made by George and Mallery [35] and Nunnally [36] with respect to having values of 0.6 (questionable analysis) or 0.5 (poor values). This means that the research process can be continued with values of 0.5, considering that for the next phase, the intentions and effects of each variable that integrates a factor or block should be reviewed [37]. The formula with which the alpha value is calculated is shown below:

$$a=\frac{K}{K-1}\left[1-\frac{{\sum S}_{i}^{2}}{S_{T}^{2}}\right]$$

where:

K: the number of items

Si2: variance of the sum of the items

St2: sum of the variances of the items

α: Cronbach’s alpha coefficient

## Results

Regarding the analysis of the effect of skills on organizational performance mediated by the dynamic capacity for innovation in MSMEs located in Caquetá, in the first scenario, we identify the reliability of the instruments examined via the software, which yield a value of 0.971 for the Cronbach’s alpha and a value of 0.983 for the RHO reliability coefficient. These values indicate adequate internal consistency of the instruments, as shown in Table 4.

**Table 4:** Analysis of the internal consistency and convergent validity of the model.

Construct	dimension	Indicator	Robust T	CF > 0.6 factor loading Squared factor	loading Mean factor loading	Error	Average error	Cronbach’s Alpha > a 0.7	IFC > a 0.7 composite reliability index	IVE > a 0.5, variance extracted index	Construct
Management skills	Conceptual skills (F1)	HG6	1.000	0.737	0.543	0.785	0.457	0.375	0.882	0.892	0.625
		HG9	14, 310	0.631	0.398		0.602				
		HG12	20, 321	0.869	0.755		0.245				
		HG15	19, 990	0.856	0.733		0.267				
		HG18	19.409	0.834	0.696		0.304				
		Σ		*3*.*927*	*3*.*125*		*1*.*875*				
	Technical skills (F2)	HG1	1.000	0.810	0.656	0.806	0.344	0.342	0.915	0.919	0.658
		HG4	15.090	0.621	0.386		0.614				
		HG7	23.868	0.871	0.759		0.241				
		HG10	20.312	0.781	0.610		0.390				
		HG13	23.972	0.874	0.764		0.236				
		HG16	24.149	0.878	0.771		0.229				
		Σ		*4*.*835*	*3*.*945*		*2*.*055*				
	Human skills (F3)	HG2	1.000	0.787	0.619	0.810	0.381	0.340	0.913	0.913	0.660
		HG5	21.246	0.830	0.689		0.311				
		HG8	19.861	0.789	0.623		0.377				
		HG11	23.442	0.891	0.794		0.206				
		HG14	22.005	0.852	0.726		0.274				
		HG17	17.417	0.712	0.507		0.493				
		Σ		*4*.*861*	*3*.*958*		*2*.*042*				
Dynamic innovation capacity	Technology options (F4)	CDI1	1.000	0.733	0.537	0,776	0.463	0.391	0.894	0.902	0.609
		CDI2	18.919	0.839	0.704		0.296				
		CDI3	17.194	0.767	0.588		0.412				
		CDI4	18.863	0.836	0.699		0.301				
		CDI5	19.650	0.869	0.755		0.245				
		CDI6	13.510	0.611	0.373		0.627				
		Σ		*4*.*655*	*3*.*657*		*2*.*343*				
	Conceptualization (F5)	CDI7	1.000	0.865	0.748	0.843	0.252	0.289	0.880	0.880	0.711
		CDI8	23.149	0.813	0.661		0.339				
		CDI9	25.030	0.850	0.723		0.278				
		Σ		*2*.*528*	*2*.*132*		*0*.*868*				
	Coproduce (F6)	CDI10	1.000	0.742	0.551	0.798	0.449	0.361	0.775	0.779	0.639
		CDI11	18.479	0.853	0.728		0.272				
		Σ		*1*.*595*	*1*.*278*		*0*.*722*				
	Scale (F7)	CDI12	1.000	0.855	0.731	0.846	0.269	0.284	0.881	0.883	0.716
		CDI13	25.336	0.879	0.773		0.227				
		CDI14	21.822	0.803	0.645		0.355				
		Σ		*2*.*537*	*2*.*148*		*0*.*852*				
Organizational performance	Internal processes (F8)	DO1	1.000	0.853	0.728	0.886	0.272	0.214	0.916	0.917	0.786
		DO2	27.377	0.908	0.824		0.176				
		DO3	26.870	0.898	0.806		0.194				
		Σ		*2*.*659*	*2*.*358*		*0*.*642*				
	Open system (F9)	DO4	1.000	0.869	0.755	0.871	0.245	0.242	0.904	0.904	0.758
		DO5	25.909	0.865	0.748		0.252				
		DO6	26.636	0.878	0.771		0.229				
		Σ		*2*.*612*	*2*.*274*		*0*.*726*				
	Rational model (F10)	DO7	1.000	0.851	0.724	0.895	0.276	0.198	0.923	0.924	0.802
		DO8	27.556	0.914	0.835		0.165				
		DO9	27.868	0.920	0.846		0.154				
		Σ		*2*.*685*	*2*.*406*		*0*.*594*				
	Human relations (F11)	DO10	1.000	0.893	0.797	0.774	0.203	0.393	0.849	0.820	0.607
		DO11	17,261	0.690	0.476		0.524				
		DO12	19.044	0.739	0.546		0.454				
		Σ		*2*.*322*	*1*.*820*		*1*.*180*				

S-BX2 (df = 903) = 6874.684; p < 0.0000; NFI = 0.757; NNFI = 0.852; CFI = 0.862; RMSEA = 0.044.

Legend: ^a^ = Parameters constrained to that value in the identification process.

*** = p < 0.001.

Source: Authors.

**Fig 2:**
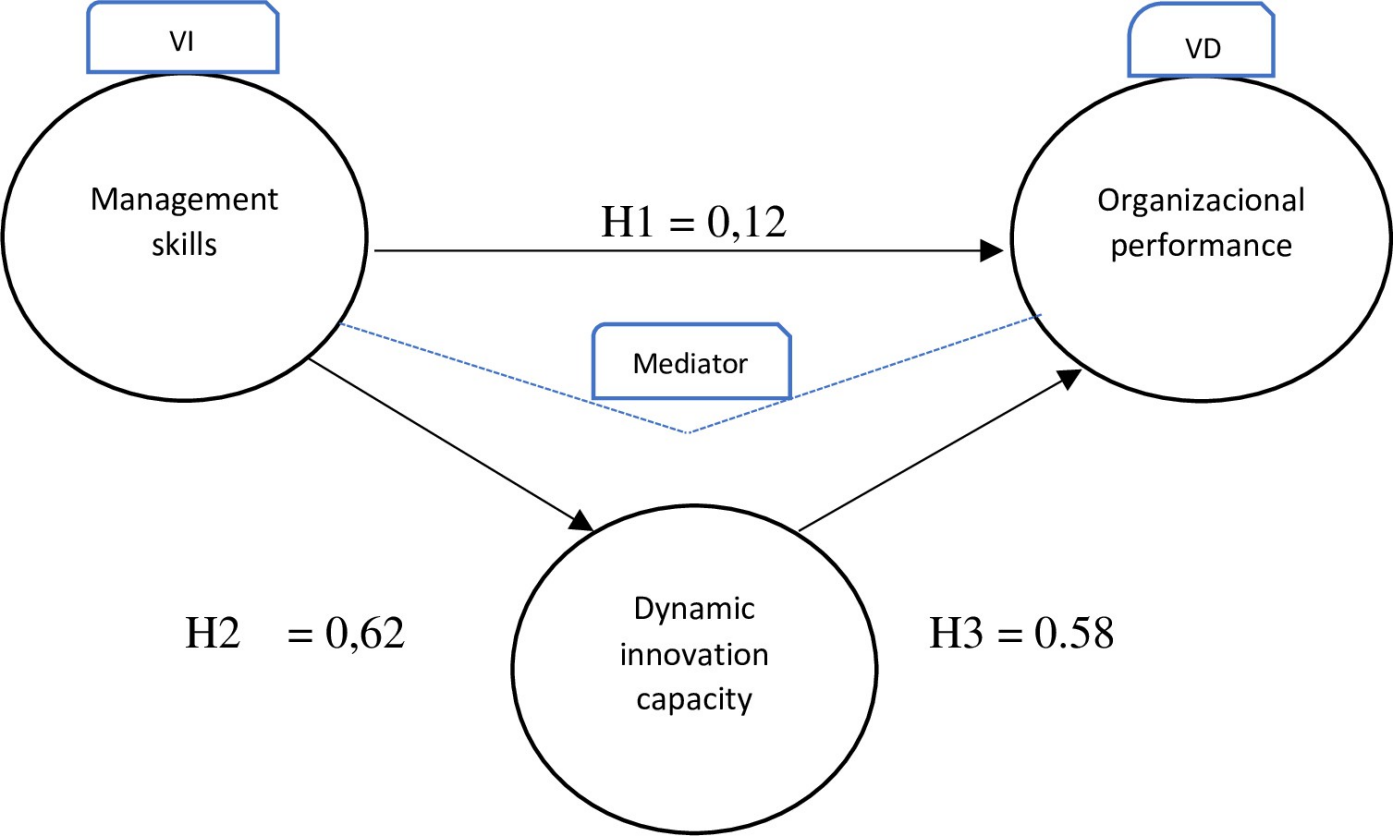
Effect from the regression equation involving the three variables. Source: Authors.

The robust fit values obtained for this model are an RMSEA of 0.044 and a CFI of 0.862. Both values meet the requirements to conclude that the model has a good fit and is therefore reliable, as shown in Fig 2.

In the model, we find greater significance in the correlation coefficients, which indicates that the dynamic innovation capability variable plays a total mediation role between managerial skills and organizational performance.

To verify that the mediation exerted by the dynamic innovation capability variable is total, it is necessary to implement the Sobel test, as shown in Table 5.

**Table 5:** Results of the mediation effects.

Hypotheses Standardized coefficients Statistical Z test Standardized error	Hypotheses Standardized coefficients Statistical Z test Standardized error	Hypotheses Standardized coefficients Statistical Z test Standardized error	Hypotheses Standardized coefficients Statistical Z test Standardized error
H2: HG → CDI	0.619***	6.59	0.061
H3: CDI → DO	0.665***	8.02	0.078
H4: HG → CDI→ DO	0.412***	6.527	0.063

Legend: n = 496. To calculate the standardized coefficients in this table, two models are calculated. The first model calculates the standardized coefficient of the HG-CDI (S-Bχ2 = 6874.684 [df = 903]; p < 0.0000; CFI = 0.862; NNFI = 0.852; IFI = 0.757; RMSEA = 0.044)). The second model is used to calculate the standardized coefficient of the CDI-DO (S-Bχ2 = 1148.054 [df = 426]; p < 0.0000; CFI = 0.906; NNFI = 0.898; IFI = 0.907; RMSEA = 0.041)). The coefficients used to calculate the indirect effects of the HG-CDI-DO are as follows: S-Bχ2 = 2263.943 [df = 846]; p < 0.0000; CFI = 0.862; NNFI = 0.852; IFI = 0.863; RMSEA = 0.044. Fit indices, Z test statistics, and standard errors for main effects are estimated via the robust maximum likelihood (MLR) method. Z test statistics and p values for indirect effects are estimated with the Sobel test. Notation: ^↑^p < .10 * p < .05 ** p < .01

*** p < .001.

Source: Authors.

With the hypothesis tests performed above, it is also possible to informally review the mediation generated by the dynamic innovation capacity variable. To examine such mediation, it is sufficient to verify the following four conditions:

1. The independent variable significantly affects the mediator;
2. The independent variable significantly affects the dependent variable in the absence of the mediator;
3. The mediator has a significant effect on the dependent variable; and
4. The effect of the independent variable on the dependent variable is reduced with the addition of the mediator to the model.

Condition 1 is met as stated in Hypothesis 2, where the effects between the independent variable (managerial skills) and the mediator (dynamic innovativeness) are evaluated. On the basis of the results, the significance is verified (p < 0.05), the sizes of all factor loadings on each indicator are greater than 0.6, the index of variance extracted from each pair of constructs is greater than 0.5, and the Cronbach’s alpha of each construct is greater than 0.7. These derivations are concurrent with those of previous studies, such as those of Laud et al. [12], Lepak and Snell [14] and Woodruffe [15], who suggest that managerial skills have a positive effect on the dynamic capacity for innovation in MSMEs.

The internal factor of the managerial skills of the managers of MSMEs has a significant influence on organizational performance, which is reflected in the findings of Leyva et al. [38]. Herein, we show the factor loadings that highlight the shared variance between the managerial skills construct and its respective indicators (strategic management, strategic planning, globalization and human resources), which are related to the study of managerial skills via the instrument developed by Northouse [8]. Taking as a study sample the managers of the 496 examined MSMEs located in the department of Caquetá, when performing the analysis according to the study dimensions related to managerial skills, the indicators (growth strategy, functional detail of things, continuous work among all integrated parts) predominate.

According to the analysis, the factor load of HG3, which is part of the managerial skills construct in the conceptual skills variable, is below 0.6 and is thus low, with a value of 0.315. This is related to the fact that all the people who responded to the survey saw this variable in one of either two ways, namely, they did not understand the question or they did not like to work with abstract ideas. Although the importance of this variable as a managerial skill is recognized in the literature, the applied model made it possible to establish its low degree of participation in the management of MSMEs located in the department of Caquetá.

The design of strategies focuses on generating greater competitive advantage. According to López [39], management skills are based on both mechanism and technique, which function integrally within the organization and are integrated into different contexts, thereby allowing business management on the basis of the combination of experience and working with human capital. Therefore, it becomes a functional component of integration with dynamic capacity in organizations.

The impact of the dynamic capacity for innovation in SMEs is relevant for adapting to changes in the environment [40]. This leads to the use of a methodology in organizations with the participation of managers; in this scenario, organizational skills are representative, which allows the development and reconfiguration of dynamic capabilities and their interaction with the environment [41].

Managerial skills have a positive effect on dynamic capabilities. Dynamic managerial capability theory analyzes how managers make strategic decisions to help build and maintain competitive advantage in dynamic environments [42]. Organizational learning and strategic leadership play crucial roles in the development of dynamic capabilities [43].

The relationship between managerial skills and the dynamic capacity for innovation is explored in Model 2, in which a CFI of 0.861 is obtained, which represents a reasonable fit of the model; this is corroborated by the RMSEA, whose result is 0.044, indicating a good fit since it is below the threshold of 0.05. On the basis of the results, the significance of the related values is verified (p < 0.05), the sizes of all the factor loadings for each indicator are greater than 0.6, the index of variance extracted from each pair of constructs is greater than 0.5, and the Cronbach’s alpha of each construct is greater than 0.7.

Condition 2 is fulfilled according to the results presented in which the effects between the independent variable (managerial skills) and the dependent variable (organizational performance) are evaluated without mediation. The Cronbach’s alpha value obtained is 0.61; therefore, the reliability of the instruments applied and the proposed items is verified. We also revied the reliability coefficient RHO, which results in a value of 0.97, which, being very close to 1, guarantees the internal consistency of the instruments applied. This analysis indicates that managerial skills have a direct and significant influence on organizational performance, with results that coincide with the findings of studies such as Singh et al. [9], who state that management skills have a direct and significant influence on organizational performance, thereby highlighting the ability of strategic leadership, which is an inherent skill for digital transformation and innovation, specifically in basic management activities [9].

The investigation by Coaquira [6] refers to transformational leadership and knowledge management, which are variables that influence organizational performance and represent an indirect and positive effect, thus generating a relationship of quality management-knowledge management-organizational performance and quality management-transformational leadership-organizational performance.

Concerning the discussion of the results, it is necessary to highlight those mentioned by Coaquiera [6], who refers to the development of quality management practices that are not only oriented to the customer and continuous improvement but also serve as support in the processes of knowledge management and leadership. These findings can be applied to MSMEs located in the department of Caquetá, especially to companies in the service sector.

Condition 3 is fulfilled given that management skills have a positive effect on the dynamic capacity for innovation in MSMEs located in Caquetá. This shows that management skills have a positive effect on the dynamic capacity for innovation in MSMEs located in Caquetá.

Finally, for Condition 4, we compare the effects between the independent variable and the dependent variable with and without mediation. Managerial skills have a positive influence on the organizational performance of MSMEs located in the department of Caquetá; the incidence rate is 0.49 for the effects between managerial skills and organizational performance when there is no mediator, whereas Fig 1 shows a value of 0.12 for the effects between the same variables when there is mediation of dynamic innovation capacity.

The latter condition also verifies the significance values, with a *Z value > 1*.*96* used as the validation criterion. The results reveal that dynamic innovation capability is significant *(B = 7*.*209; p < 0*.*01*), whereas the significance of organizational performance is considerably reduced *(B = 1*.*847; p < 0*.*01)* when it is affected by a mediator. We infer, therefore, that the mediation of dynamic innovativeness is of the total type in the model.

Given that all 4 conditions are met, we can state that the dynamic innovativeness variable has mediating effects in that it significantly influences the effects of the dependent and independent variables. Therefore, Sobel’s test supports Hypothesis 4 insofar as it explains the mediating nature of dynamic innovativeness in terms of the effects of managerial skills on organizational performance.

Table 6 verifies the four hypotheses and shows the results, their influence and their incidence rate.

**Table 6:** Summary of hypotheses.

Hypothesis Expected sign Results	Hypothesis Expected sign Results	Hypothesis Expected sign Results
H1: Management skills positively influence organizational performance.	*+*	Have a direct and significant influence
H2: Management skills have a positive impact on the dynamic capacity for innovation.	*+*	Have a positive impact
H3: Dynamic innovation capacity positively affects organizational performance.	*+*	Has a positive impact
H4: Dynamic innovation capacity mediates the relationship between management skills and organizational performance of MSMEs.	*+*	Mediation significantly influences

Source: Authors.

## Conclusions

The lack of planning and the development of competitive advantage have a negative influence on the business environment of SMEs in the business development of MSMEs; the influence of variables such as management skills and their influence on business development is evident. "Technical" skills have a significant influence on the functioning of the different processes and procedures carried out in organizations; likewise, the human skills dimension is an indicator of greater influence on teamwork and group dynamics. According to the characterization of the different variables of the dimensions of management skills studies, the importance of aspects such as leadership and managerial skills in the management of work teams is established. The study variable of organizational performance should be integrated into four dimensions: internal processes, the open system, the rational model and human relations. Dynamic capabilities (innovation) enable companies to create, implement and protect intangible assets that support long-term business performance.

Comparing the effects of managerial skills to unmediated organizational performance (H1) with the effects of managerial skills to mediated organizational performance (H3) better explains the effects of managerial skills on organizational performance when mediated by the dynamic capability of innovation.

The significant contribution of the present research is the study of the dynamic capacity for innovation within MSMEs; herein, this factor is used as a mediating variable that allows the analysis of managerial skills and the development of activities that can be verified and evidenced. In this way, the current study provides recommendations that are useful for companies that are legally constituted through the Chamber of Commerce of Florencia in Caquetá at the moment of implementing strategies oriented toward management skills, organizational performance and dynamic innovation capabilities.

To this end, it is necessary for entrepreneurs to be aware of the relevant points that must be established and logically improved to orient their efforts in the right direction. In turn, companies that assume the responsibility of including the dynamic capacity for innovation as a form of exploration tend to improve their capabilities, increasing the possibility of creating a competitive advantage that is more in line with the company’s business and strengthening its current needs, which enables the creation of greater value.

Our results are relevant for entrepreneurs and innovation managers in MSMEs who can find valuable guidance on how to strengthen innovation performance under the nexus between the dynamic capabilities perspective and managerial skills, with a focus on both organizational and managerial aspects.

## Limitations

The main limitations evidenced in the development of this study are related to the region where the research was carried out, where there are few business dynamics and little permanence of the same in the market, resulting in inaccurate results over a prolonged period for the analysis of the business development dynamics of the sectors. In addition, many businessmen, managers or directors refrained from providing information.

## Future lines of research

The theory of dynamic capabilities is based on concepts such as organizational learning, knowledge absorption, innovation, added value, adaptability, business competitiveness, internationalization and survival, which may be viable for the business study of MSMEs located in the department of Caquetá.

In the future, we aim to develop this study approach in other areas of the country (and internationally) as a mechanism that contributes to business development in emerging economies, especially in larger companies.

We hope that the study herein has established a work methodology through which the MSMEs surveyed can learn how their work is being carried out and how—on the basis of the development of capacities—they can achieve greater performance and growth in the region.

## Supporting information

S1 Fig Tfp140623.EIS SEM model, built using Structural Equations Program EQS version 6.4 for Windows. by Peter M. Bentler and Eric J.C. Wu. Distributed by Multivariate Software, S.A.P.I de C.V. (EIS)
